# Seroprevalence of viral pathogens associated with bovine respiratory disease complex and biosecurity-related risk factors in cattle farms in Türkiye

**DOI:** 10.1007/s11250-026-04869-2

**Published:** 2026-01-20

**Authors:** Yavuzkan Paksoy, Ömer Barış İnce, Emrah Gökay Özgür, Ahmet Sait

**Affiliations:** 1 Department of Animal Nutrition and Animal Husbandry, Ceyhan Faculty of Veterinary Faculty, Çukurova University, Adana, Türkiye; 2 Department of Virology, Veterinary Faculty, Necmettin Erbakan University, Eregli, Konya 42310 Türkiye; 3 Department of Biostatistics and Medical Informatics, Faculty of Medicine, Marmara University, Istanbul, Türkiye; 4 Viral Diagnostic Laboratory, Pendik Veterinary Control Institute, Pendik, Istanbul, 34890 Türkiye

**Keywords:** Bovine respiratory disease complex, Biosecurity, Cattle, Viral pathogens, Risk factors

## Abstract

Bovine respiratory disease complex (BRDC) is one of the main causes of illness and financial losses in cattle farming around the world. The aim of this cross-sectional study was to estimate the seroprevalence of viral pathogens associated with the bovine respiratory disease complex (BRDC) and to identify farm- and animal-level risk factors, including biosecurity practices and indirect contacts, in cattle farms in Konya and Adana provinces of Türkiye. However, the role of farm-level biosecurity practices and indirect contacts in shaping these infection patterns remains unclear, including in Türkiye where data on farm-level biosecurity practices and indirect contacts are limited. This cross-sectional study involved collecting blood and nasal swab samples from 274 cattle on 16 farms in the provinces of Konya and Adana between March 2023 and April 2024. Blood serum samples were tested using indirect ELISA to determine the presence of antibodies against Bovine respiratory syncytial virus (BRSV), Bovine parainfluenza virus-3 (BPIV-3), and Bovine viral diarrhea virus (BVDV). Nasal swabs were analyzed using one-step multiplex real-time RT-PCR to detect the presence of viral nucleic acids. Information about biosecurity at the farm level was collected using a questionnaire and field observations. Potential risk factors were evaluated using logistic regression and generalized estimating equations (GEE). At the farm level, the number of seropositive animals varied from 0% to 50%, while at the animal level, the overall seropositivity rate was 21% (57 out of 274). The test showed that 2.19% (6/274) of the samples had BVDV RNA, but none had BRSV or BPIV-3. In the multivariable analyses, increasing age (odds ratio [OR] = 7.05; 95% confidence interval [CI]: 3.21–15.48; *p* < 0.001) and farm visitors (OR = 11.74; 95% CI: 4.86–28.5; *p* < 0.001) were found to be the most significant risk factors. The size of the farm was almost statistically significant. The study showed substantial heterogeneity between farms in seropositivity for viral BRDC pathogens, and highlighted the role of indirect contacts and biosecurity-related factors in shaping these patterns. Visitors and shared transport vehicles were identified as the main determinants of transmission. Main strategies to reduce risk include providing protective clothing, minimizing unnecessary animal movements, and strengthening vehicle disinfection protocols. The findings highlight the importance of raising biosecurity awareness among farmers and veterinarians. Due to budgetary and logistical constraints, this exploratory study was conducted in a relatively small number of herds from only two provinces.

**Supplementary Information:**

The online version contains supplementary material available at https://doi.org/10.1007/s11250-026-04869-2.

## Introduction

Bovine respiratory disease complex (BRDC) is a multifactorial syndrome characterized by elevated morbidity and mortality rates, particularly among young calves and feedlot cattle (Yarnall et al. 2024; O’Donoghue et al. 2025). The BRDC is the result of a combination of viral and bacterial pathogens, environmental stress, and management practices. Previous studies have shown that BRDC can cause reduced growth rates, decreased milk production, increased treatment and labour costs, and additional workload for herd health management, leading to substantial economic losses in cattle production systems (Smith 2020; Yarnall et al. 2024). The primary viral pathogens associated with BRDC include Bovine herpesvirus-1 (BoHV-1), Bovine respiratory syncytial virus (BRSV), Bovine parainfluenza virus-3 (BPIV-3), and Bovine viral diarrhea virus (BVDV).In recent years, *the influenza D virus* (IDV) has also been considered within this group (Ferreira et al. 2024). These pathogens can spread through direct transmission (animal movements, droplet transmission) as well as indirect routes (visitors, transport vehicles, shared equipment, fomites, pastures). Although indirect contacts may appear less effective than direct contacts regarding disease transmission, their high frequency makes them an important epidemiological issue that is worthy of further investigation. Veterinarians, artificial insemination technicians, feed and transport vehicles, and even staff working at neighboring farms may act as carriers of pathogens between farms (Bates et al. 2001; Rossi et al. 2017). This situation demonstrates the necessity of considering indirect contact in farm biosecurity protocols.

Biosecurity includes management and physical measures aimed at preventing the introduction of infectious agents into farms and limiting their spread within the herds. The “prevention is better than cure” approach adopted by the European Union in 2007 emphasizes that farm-level biosecurity should be at the core of animal health strategies (Ferreira et al. 2024). However, studies conducted in various countries have shown that biosecurity practices are lacking or insufficient on many farms (Sarrazin et al. 2018; Alquati et al. 2025).

There have been various studies on the prevalence of BVDV and other respiratory viruses in Türkiye (Özkul et al. 1995; İnce 2020; Kadiroğlu et al. 2020; İnce and Ayaz 2023), but there are no comprehensive studies that look at the risk of these infections entering farms through indirect contact. This gap poses a major limitation for the development of effective biosecurity strategies at the national level.

The aim of this cross-sectional study was to estimate the seroprevalence of viral pathogens associated with the BRDC and to identify farm- and animal-level risk factors, including biosecurity practices and indirect contacts, in cattle farms in Konya and Adana provinces of Türkiye. Field data were collected using serological and molecular tests, survey-based biosecurity assessments were conducted, and risk factors were examined using statistical models. The study thus provides scientific evidence on which biosecurity measures should be prioritized on farms and offers recommendations to address gaps in implementation.

## Material and methods

### Study area and sampling design

**Fig. 1:**
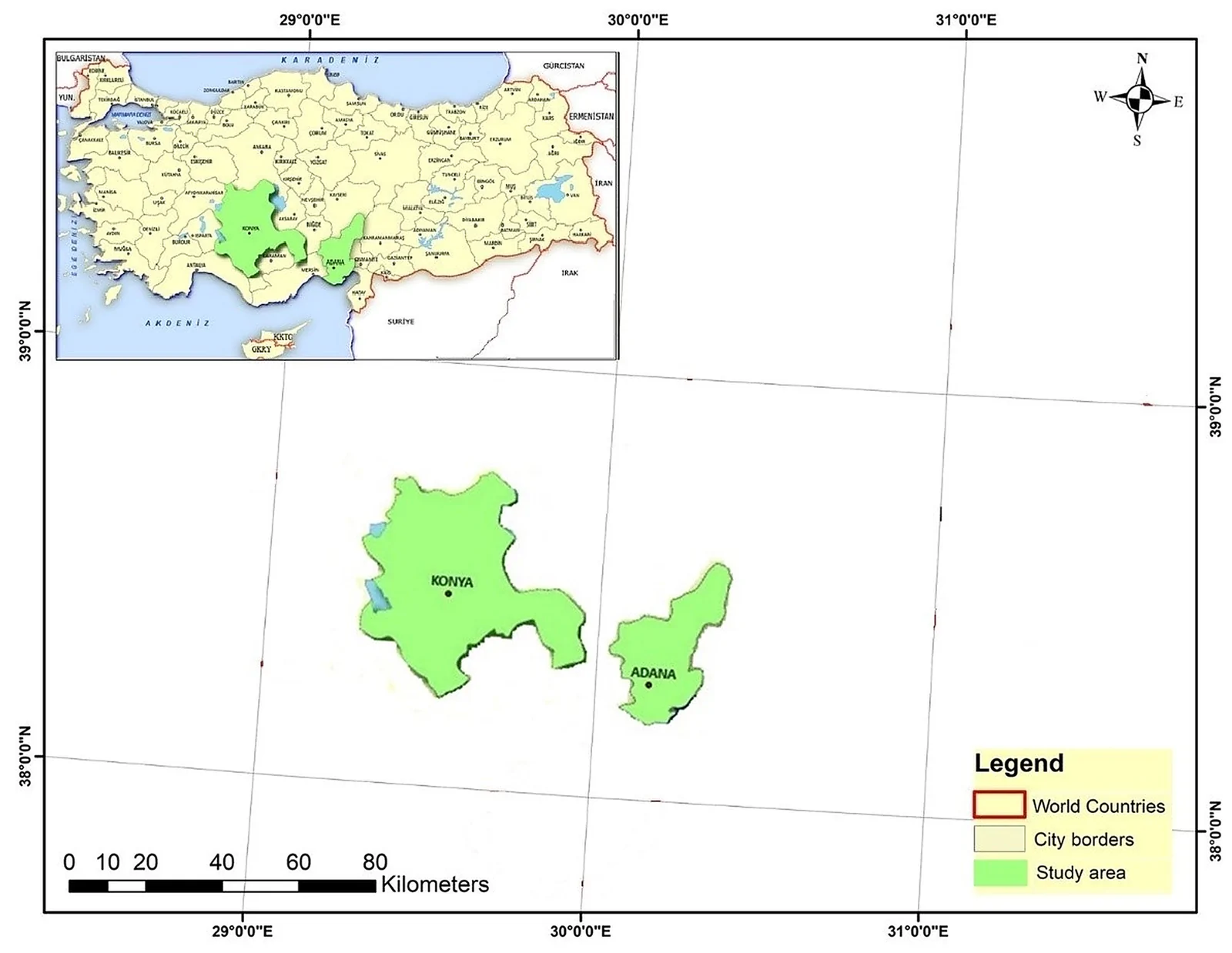
Study area

This cross-sectional study was conducted in 16 cattle farms located in the provinces of Konya (37°52′-39°08′ N; 31°14′-34°26′ E) and Adana (36°01′-37°45′ N; 34°25′-36°36′ E) between March 2023 and April 2024 (Fig. 1). The study area comprises approximately 31,000 cattle farms (TUIK 2022). The cattle farms included in the study were selected from among those that agreed to participate using Neyman’s stratified sampling method (Neyman 1934). The provinces of Konya and Adana were chosen as the study area because they are located in different regions of Türkiye with distinct climatic conditions, represent important cattle-producing areas, and provide marked diversity in farm management and biosecurity practices. The farms were considered in two strata according to province, and the total number of farms to be sampled was set at *n* = 16, taking into account field and logistical constraints. To ensure representativeness across strata, proportional allocation was applied using Neyman’s stratified sampling method (Neyman 1934):

$$\:{n}_{h}=\frac{{N}_{h}}{N}\cdot\:n$$

Where:

- n_h_ = number of farms to be sampled in stratum h (province)
- N_h_​ = total number of farms in stratum h
- N = total number of farms
- n = total sample size (number of farms)

Accordingly, a total of 16 farms were included. From these farms, individual samples were collected from 274 cattle, thus balancing the limited number of farms with a larger animal-level sample size (Cochran 1977; Neyman 1934; Dohoo et al. 2009).

### Biosecurity data

A structured questionnaire was administered to farm owners or managers to evaluate farm-level biosecurity practices and indirect contacts. The questionnaire consisted of 43 items grouped into seven sections: (i) general farm characteristics, (ii) animal movements and purchase practices, (iii) visitor access and human-related contacts, (iv) hygiene and biosecurity measures for people and equipment, (v) vehicles and environmental hygiene, (vi) herd health management and farmer perceptions, and (vii) additional perceptions and experience related to BRDC and biosecurity. Items covered general farm information, animal movements, pasture use, purchase of animals from outside sources, transport vehicles entering the farm premises, visitor frequency, and the use of protective equipment by farm personnel. Most questions were closed-ended with pre-defined response categories (e.g. yes/no, multiple choice or Likert-type scales), complemented by a small number of items allowing multiple responses and “other: please specify” options. These data were used as input variables in the analysis of potential risk factors. The questionnaire was developed, implemented and reported in line with the STROBE guidelines (von Elm et al. 2008; www.strobe-statement.org). The model structure of the biosecurity questionnaire (English translation) is presented in Supplementary Table S1, and the completed STROBE checklist is provided in Supplementary Table S2.

### Laboratory analyses

Blood samples were collected from the *jugular vein* of a total of 274 cattle into both coagulated and anticoagulated tubes and transported to the laboratory under cold chain conditions. The presence of antibodies against BRSV, BPIV-3, and BVDV in serum samples was tested using commercial ELISA kits [Bio-X Diagnostics BRSV Ab Test and BPI3 Ab Test (Rochefort, Belgium); IDEXX BVDV Total Ab Test (IDEXX Laboratories, Westbrook, ME, USA)] following the manufacturer’s instructions. Nasal swab samples were then homogenized in Phosphate Buffered Saline (PBS), following which the samples were subjected to a centrifugation process at 1200 × g for a duration of 20 min. Thereafter, the samples were stored at a temperature of -80 °C. The process of RNA extraction was conducted utilizing the QIAamp cador Pathogen Mini Kit (Qiagen, Germany). The method was optimised according to the procedures specified in the kit. Viral RNA was detected by one-step multiplex real-time RT-PCR using primers and probes specific for the BVDV 5′UTR region, the BRSV nucleoprotein gene, and the BPIV-3 matrix gene (Lindberg et al. 2006) (Table 1). The fixed reaction conditions for the multiplex system were as follows: a total reaction volume of 20 µL containing 5 µL of 5× QuantiFast Pathogen Master Mix, 0.25 µL of 100× QuantiFast Pathogen RT Mix, 0.1 µL of each primer (10 µM), 0.2 µL of each probe (10 µM), 3 µL of nucleic acid template, and RNase-free water added to a final volume of 20 µL. Amplification was performed using the following program: 50 °C for 20 min, 95 °C for 5 min, followed by 45 cycles of 95 °C for 15 s and 60 °C for 30 s.

**Table 1:** Primer and probe sequences used in One-Step Multiplex Real-Time PCR studies

Agent	Primer/prob	Sequence (5’-3’)
BVDV	BVDV-F228 BVDV-UTR BVDV-Pro	TCGAGATGCCACGTGGAC ATGTGCCATGTACAGCAGA CY5-ACCCTATCAGGCTGT-MGB
BRSV	BRSV-N-141 bp-F BRSV-N-141 bp-R BRSV-N-Probe	ATACAAAGGACTCATCCCGAAAG AAGATTCCTTCTACCCTACTACCTCC NED-AGTATTTGAAAAGTACCCTC-MGB
BPIV3	BPIV3M-113 bp-F BPIV3M-113 bp-R BPIV3-MTPro	CAGGAACTCCTACAAGCCGC CATGGGTACAGTTCAGGTTTAATG VIC-CTATCATCTCCGTGGC-MGB

### Risk factors and statistical analysis

For each farm, variables such as the animal’s age, sex, farm size, shared grazing with small ruminants, acquisition of new animals, and lactation status were considered potential risk factors. For the purposes of analysis, explanatory variables were grouped into two conceptual domains: (i) indicators of indirect contact (e.g. visitor frequency, presence of permanent workers who also worked on other farms, animal purchases, use of common pastures) and (ii) farm-level biosecurity practices (e.g. use of farm-dedicated protective equipment, recording of visitors, presence of disinfection mats and quarantine facilities). All data were coded and entered into Microsoft Excel, then transferred to the R program for analysis. Statistical analyses were performed using R version 4.5.1 (R Core Team 2025). To estimate seroprevalence, a statistical model accounting for diagnostic uncertainty, considering test sensitivity (Se) and specificity (Sp), was applied (Sergeant 2017). Descriptive statistics were presented as frequencies and percentages, while seroprevalence estimates were presented with 95% confidence intervals (CI). In univariable analyses, Pearson’s chi-square and Fisher’s exact tests were used, and variables with *p* ≤ 0.20 were included in multivariable logistic regression analysis. Relationships between positive ELISA results and risk factors were evaluated using odds ratios (OR), and the goodness-of-fit of multivariable models was assessed with the Hosmer-Lemeshow test (Dohoo et al. 2009).

## Results

### Seroprevalence

Antibodies against at least one respiratory virus were detected in 57 out of 274 serum samples from cattle (21.0%; 95% CI: 16.1–26.7) (Table 2). When evaluated by virus, BRSV had the highest seropositivity rate (8.4%). This was followed by BPIV-3 (6.6%) and BVDV (5.8%). These results indicate that viruses associated with BRDC are circulating at a certain rate in the study area. According to the generalized estimating equation (GEE) model, the overall seroprevalence within the farm was estimated to be 21% (95% CI: 16.1–26.7). At the farm level, seropositivity rates ranged from 0% to 50%. This wide variation is consistent with heterogeneity in management, biosecurity practices and environmental conditions between farms; however, the present study was not designed to disentangle these effects, and the observed differences should therefore be interpreted with caution.

**Table 2:** Antibody positivity rates detected by ELISA

Agent	Positive	Total	Seropositive (%)	95% CI
BRSV	23	274	8.4	5.4–12.4
BPIV-3	18	274	6.6	4.1–10.3
BVDV	16	274	5.8	3.5–9.4
Total	57	274	21.0	16.1–26.7

### PCR

Multiplex real-time RT-PCR analysis of nasal swab samples detected only BVDV RNA (6/274; 2.19%; 95% CI: 0.9–4.6) (Table 3). In contrast, no samples tested positive for BRSV or BPIV-3. This finding indicates that active viral infection of the respiratory system was low during the study period, but that BVDV remains in circulation in the region.

**Table 3:** Multiplex real-time RT-PCR results

Agent	Positive	Total	Positive (%)	95% CI
BVDV	6	274	2.19	0.9–4.6
BRSV	0	274	0	–
BPIV-3	0	274	0	–

### Risk factors

In univariable analyses, age, visitor presence, farm size, and new animal introduction were found to be associated with seropositivity. In the multivariable logistic regression analysis, age and visitor presence emerged as the strongest risk factors (Table 4). The risk of seropositivity increased sevenfold in cattle aged two years and older (OR = 7.05; 95% CI: 3.21–15.48; *p* < 0.001). The risk of infection was significantly increased by approximately 12-fold in farms where visits occurred (OR ≈ 11.74; 95% CI: 4.86–28.5; *p* < 0.001). In contrast, farm size showed borderline statistical significance (*p* = 0.08), while the introduction of new animals did not show a significant effect in the multivariable model.

**Table 4:** Multivariable logistic regression analysis results

Variable	Categories	OR	95% CI	*P* value
Age	06–12 months*			
	> 12 months	7.05	3.21–15.48	< 0.001
Farm size	≤ 20*			
	21–100	2.280	0.524–9.918	0.109
Farm visitors	No*			
	Yes	11.74	4.86–28.5	< 0.001

* References

OR: Odds ratio

CI: Confidence interval

## Discussion

In this cross-sectional study, we estimated the seroprevalence of viral pathogens associated with the BRDC in cattle farms located in the Konya and Adana provinces of Türkiye and evaluated their association with selected farm- and animal-level risk factors, including biosecurity practices and indicators of indirect contact. The overall seroprevalence was 21.0%, which lies at the lower end of the wide range of values reported in Türkiye and worldwide. These relatively low seroprevalence levels may reflect a combination of factors, including genuinely lower infection pressure in the study area, partial effectiveness of certain biosecurity measures implemented on some farms, and the limited number of herds and animals included in this exploratory study. In this study, BRSV seropositivity was determined to be 8.4%. This rate is significantly lower than the 46–73% range previously reported in Türkiye (Yavru et al. 2005; Yeşilbağ and Güngör 2008; Karaotçu and Yıldırım 2019; Yıldırım et al. 2009; Kadiroğlu et al. 2020; İnce et al. 2022; Aydın et al. 2024). In the international literature, seroprevalence estimates range from around 30% (e.g. Argentina, Sweden) to 80% (e.g. Brazil, Ecuador) (Hägglund et al. 2006; Saa et al. 2012; Hoppe et al. 2018). The relatively low estimate observed in our study may reflect a combination of factors, including sample size, age distribution, and specific management and biosecurity practices in the participating herds. A recent meta-analysis has reported a global average BRSV seroprevalence of 62% based on antibody-based tests (Werid et al. 2024), placing our findings at the lower end of both the national and international ranges and suggesting that infection pressure may be comparatively limited in the study area, although this interpretation should be made with caution given the limited number of herds.

Seropositivity was higher in adult cattle than in young animals, which is consistent with the role of repeated lifetime exposure in the development of humoral immunity. Similar age-related patterns, with higher BRSV seroprevalence in adults, have been reported in studies from other countries (Figueroa-Chávez et al. 2012; Hoppe et al. 2018). The seroprevalence of BPIV-3 has been determined to be 6.6%. Studies in Türkiye have generally reported rates between 10 and 25% (Alkan et al. 2003; Erol et al. 2007; Yeşilbağ and Güngör 2008; Kadiroğlu et al. 2020), while global estimates range from 15% to 70% (Ellis 2010; Mahmoud and Allam 2013; Callaby et al. 2016; Tiwari et al. 2016; Muftuoglu et al. 2021; Milićević et al. 2024; Xu et al. 2024; González et al. 2025). The relatively low level observed in our study may reflect similar factors to those discussed for BRSV and BVDV, including differences in age structure and management/biosecurity practices between herds, regional variation in infection pressure, and potential differences in diagnostic sensitivity between test methods. Nevertheless, BPIV-3 clearly remains an important factor to consider in the pathogenesis of BRDC.

BVDV seropositivity was detected at 6.2%, which is lower than the 20–60% range reported in Türkiye (İnce 2020; İnce and Ayaz 2023). This may be related to relatively limited animal movements or effective control practices in some herds, but the cross-sectional design and limited sample size preclude firm conclusions. The literature indicates that, in general, one of the most important risk factors for BVDV introduction is the purchase of animals from herds that are not certified BVDV-free, which can lead to rapid spread of infection through the introduction of persistently infected animals into the farm (Bisschop et al. 2025). In a recent study, Bisschop et al. (2025) reported higher infection risk in dairy herds located within 500 m of other farms. In addition, previous studies have shown that housing structure and human-related movements can influence BVDV infection risk. In our study, the observed association between visitor and vehicle entry, the presence of permanent workers and BVDV seropositivity is consistent with this evidence. Permanent staff may work on more than one farm or have additional off-farm jobs; if protective clothing, boots and hand hygiene are not strictly standardised between farms, they may act as mechanical carriers (fomites) of pathogens. Furthermore, familiarity with daily routines may lead to “fatigue” or lapses in adherence to biosecurity protocols over time, further contributing to the observed association. Despite the low seroprevalence detected in this study, BVDV continues to be present in the region. Control of animal movements, training of farm staff in consistent biosecurity practices and avoidance of mixed-source animal purchases therefore remain important for reducing the risk of BVDV introduction and spread. In multivariable logistic regression analysis, the presence of visitors increased the risk of infection by approximately 12 times (OR = 11.74). While this strongly supports the role of visitors as carriers, it should be noted that such high OR values may also be partly explained by confounding factors. Larger herds accepting more visitors and being more vulnerable in terms of biosecurity may explain this effect (Dohoo et al. 2009; Rossi et al. 2017). Farm size showed only a trend-level association, possibly due to limited sample size.

The most important limitation of this study is the small number of herds included; this restricts the representativeness of the farm sample at regional and national level and reduces the precision of farm-level estimates. Due to field and laboratory cost constraints, this study should be regarded as exploratory/pilot in scope. Although biological samples were obtained from a total of 274 animals and adequate statistical precision was achieved at the animal level, the cross-sectional design does not allow conclusions to be drawn about temporal dynamics or causal relationships. Furthermore, detailed information on vaccination against BRDC-associated viruses and systematic recording of clinical BRDC cases were not consistently available across all farms and were therefore not included in the analysis. As a result, the seropositivity reported here should be interpreted as evidence of past exposure (due to infection and/or vaccination, where applicable) rather than as a direct measure of clinical disease occurrence at herd level. Therefore, the relatively low seroprevalence levels observed here should be regarded as exploratory findings that may reflect a combination of lower infection pressure in the study area, differences in biosecurity practices between farms, and sampling variability. Another limitation is that only three viral agents (BRSV, BPIV-3 and BVDV) were examined. Other BRDC-associated viruses such as bovine herpesvirus-1 (BoHV-1), bovine coronavirus and bovine adenoviruses, which are known to be important respiratory pathogens in cattle, were not included in the present panel due to resource constraints and the exploratory nature of the study. Future research in Türkiye should therefore consider multi-centre, larger-scale studies encompassing a broader range of herds with different levels of biosecurity measures and a wider panel of respiratory viruses, including BoHV-1, bovine coronavirus, adenoviruses and IDV, in order to confirm and refine these exploratory findings and to more robustly characterise the associations between biosecurity practices and infection risk.From a practical point of view, our findings underline the central role of everyday biosecurity practices in the prevention of BRDC on cattle farms. Measures that are likely to be particularly effective include restricting and recording visitor access to animal areas, providing dedicated protective clothing, boots and gloves for visitors and staff, strengthening cleaning and disinfection procedures at farm entrances and for vehicles entering and leaving the premises, and avoiding unnecessary animal movements and mixed-source purchases. Although the present study cannot quantify the individual effect of each of these measures, it supports the view that routine, consistently implemented biosecurity protocols are essential components of BRDC prevention.

## Conclusion

In conclusion, this exploratory study documents relatively low seroprevalence levels of major viral BRDC pathogens in cattle herds in Konya and Adana, but identifies clear associations between infection and specific biosecurity-related risk factors such as visitor presence and herd characteristics. These results suggest that strengthening basic biosecurity practices—particularly control of visitor access, consistent use of farm-dedicated protective equipment, effective cleaning and disinfection of farm entrances and vehicles, and prudent management of animal movements—can contribute to reducing the risk of introduction and spread of BRDC-associated viruses in cattle farms. Larger, multi-centre studies including more herds with varying levels of biosecurity measures are warranted to confirm and refine these recommendations. 

## Supplementary Information

Below is the link to the electronic supplementary material.


Supplementary Material 1



Supplementary Material 2


## Data Availability

The data that support the findings of this study are available from the corresponding author upon reasonable request.
