# Supplementary material for: Seroprevalence of viral pathogens associated with bovine respiratory disease complex and biosecurity-related risk factors in cattle farms in Türkiye
Source: Trop Anim Health Prod. 2026 Jan 20;58(2):56. doi: 10.1007/s11250-026-04869-2 (PMC12819427; doi:10.1007/s11250-026-04869-2)
Supplement: Supplementary file 1 — Supplementary Material 1 [file 11250_2026_4869_MOESM1_ESM.docx]

**Seroprevalence of viral pathogens associated with bovine respiratory disease complex and biosecurity-related risk factors in cattle farms in Türkiye**

**Farm biosecurity and indirect contact questionnaire used in this study**
*(Originally administered in Turkish; English translation provided below.)*

| **Section** | **Item No.** | **Item (English translation)** | **Response format** |
| --- | --- | --- | --- |
| **A. General farm characteristics** |  |  |  |
| A1 | Q1 | What is the main production type of your farm? | Multiple choice (dairy / beef / mixed / other) |
| A2 | Q2 | How many cattle are currently present on the farm? | Open numeric (number of animals) |
| A3 | Q3 | What is the predominant housing system? | Multiple choice (tie-stall / free-stall / paddock / pasture-based / other) |
| A4 | Q4 | What is the main management system? | Multiple choice (intensive / semi-intensive / extensive) |
| A5 | Q5 | Are other livestock species (e.g. sheep, goats, buffalo) kept on the same farm? | Yes/No |
| A6 | Q6 | Do cattle from different age groups share the same pens or housing areas? | Yes/No |
| **B. Animal movements and purchase practices** |  |  |  |
| B1 | Q7 | Have you purchased cattle from other farms in the last 12 months? | Yes/No |
| B2 | Q8 | If yes, what is the most common source of purchased animals? | Multiple choice (local farms / livestock markets / dealers/traders / other) |
| B3 | Q9 | Do you introduce animals from more than one source at the same time (mixed-source purchases)? | Yes/No |
| B4 | Q10 | After arrival, are newly purchased animals kept in a separate quarantine area before mixing with the herd? | Yes/No |
| B5 | Q11 | If quarantine is used, what is the usual quarantine duration? | Multiple choice (<7 days / 7–14 days / >14 days) |
| B6 | Q12 | Do your animals leave the farm temporarily for grazing on common pastures or communal areas? | Yes/No |
| B7 | Q13 | Do your animals regularly attend cattle fairs or markets (e.g. for sale, exhibition)? | Yes/No |
| **C. Visitor access and human-related contacts** |  |  |  |
| C1 | Q14 | Are visitors allowed to enter animal housing areas (barns, pens)? | Yes/No |
| C2 | Q15 | Are visitor entries to the farm recorded (e.g. in a visitor logbook)? | Yes/No |
| C3 | Q16 | How often do veterinarians visit your farm on average? | Multiple choice (at least once a week / at least once a month / a few times per year / rarely or never) |
| C4 | Q17 | How often do other professionals (e.g. AI technicians, hoof trimmers, nutrition advisors) visit your farm? | Same scale as Q16 |
| C5 | Q18 | How often do animal traders/dealers visit your farm? | Same scale as Q16 |
| C6 | Q19 | Are there permanent workers (employees working regularly on this farm)? | Yes/No |
| C7 | Q20 | If yes, do any permanent workers also work on other farms? | Yes/No/Do not know |
| C8 | Q21 | Have farm staff received any formal training on biosecurity practices in the last 3 years? | Yes/No |
| **D. Hygiene and biosecurity measures (people & equipment)** |  |  |  |
| D1 | Q22 | Are farm-dedicated boots provided for workers? | Yes/No |
| D2 | Q23 | Are farm-dedicated boots provided for visitors (e.g. veterinarians, traders)? | Yes/No |
| D3 | Q24 | Are farm-dedicated protective overalls/coveralls provided for workers? | Yes/No |
| D4 | Q25 | Are single-use or reusable gloves routinely used when handling animals? | Multiple choice (always / often / sometimes / rarely / never) |
| D5 | Q26 | Is there a hand-washing or hand-disinfection facility available at the entrance of the animal housing area? | Yes/No |
| D6 | Q27 | Is there a footbath or disinfection mat at the entrance of the animal housing area? | Yes/No |
| D7 | Q28 | Are tools and equipment used for animal handling (e.g. dehorning tools, nose tongs, syringes) used only on this farm? | Yes/No |
| D8 | Q29 | If equipment is shared with other farms, is it cleaned and disinfected before use on your animals? | Yes/No/Not applicable |
| **E. Vehicles and environmental hygiene** |  |  |  |
| E1 | Q30 | Do vehicles transporting animals (your own or external) regularly enter the farmyard or animal housing areas? | Yes/No |
| E2 | Q31 | Is there a designated area where animal transport vehicles are loaded/unloaded, separated from the main animal housing? | Yes/No |
| E3 | Q32 | Are animal transport vehicles cleaned and disinfected before loading animals at your farm? | Yes/No/Do not know |
| E4 | Q33 | Is there any wheel disinfection (e.g. wheel bath, spraying) at the farm entrance? | Yes/No |
| E5 | Q34 | How often are manure and slurry removed from animal housing areas? | Multiple choice (daily / several times per week / once per week / less often) |
| E6 | Q35 | How are dead animals (carcasses) usually disposed of? | Multiple choice (official rendering / burial on farm / disposal in open area / other) |
| **F. Farm health management and perceptions (optional short scale)** |  |  |  |
| F1 | Q36 | In your opinion, how important is BRDC as a health problem in your herd? | 5-point Likert (1 = not important, 5 = very important) |
| F2 | Q37 | How confident are you that your current biosecurity practices are sufficient to prevent introduction of respiratory diseases? | 5-point Likert (1 = not confident, 5 = very confident) |
| F3 | Q38 | To what extent do you agree with the statement: “Visitors may bring infectious agents into my farm”? | 5-point Likert (strongly disagree – strongly agree) |
| F4 | Q39 | To what extent do you agree with the statement: “Improving biosecurity is economically worthwhile for my farm”? | 5-point Likert (strongly disagree – strongly agree) |
| **G. Additional perceptions and experience** |  |  |  |
| G1 | Q40 | In the last 3 years, have you had any clinically suspected or laboratory-confirmed cases of BRDC in your herd? | Yes/No/Not sure |
| G2 | Q41 | Do you feel that maintaining or improving your current biosecurity measures creates additional cost and workload for your farm? | 5-point Likert (1 = strongly disagree, 5 = strongly agree) |
| G3 | Q42 | What are your main sources of information on infectious cattle diseases and biosecurity? (Multiple answers allowed) | Multiple choice (private veterinarian / official veterinary services / producer or farmer associations / other farmers / internet & social media / university or research institutions / other: please specify) |
| G4 | Q43 | Who is primarily responsible for ensuring biosecurity on your farm? | Multiple choice (farm owner / farm manager / veterinarian / farm workers / all of the above equally / other: please specify) |
