# Supplementary material for: Seroprevalence of viral pathogens associated with bovine respiratory disease complex and biosecurity-related risk factors in cattle farms in Türkiye
Source: Trop Anim Health Prod. 2026 Jan 20;58(2):56. doi: 10.1007/s11250-026-04869-2 (PMC12819427; doi:10.1007/s11250-026-04869-2)
Supplement: Supplementary file 2 — Supplementary Material 2 [file 11250_2026_4869_MOESM2_ESM.doc]

**STROBE Statement**—Checklist of items that should be included in reports of ***cross-sectional studies***

| **Manuscript information: Title:** **Seroprevalence of viral pathogens associated with bovine respiratory disease complex and biosecurity-related risk factors in cattle farms in Türkiye**  As the checklist was provided upon revised submission, the page number/line number reported may be changed due to copyediting and may not be referable in the published version. In this case, the section/paragraph may be used as an alternative reference. |
| --- |

| **Section/item** | Item No | Recommendation | **Reported on Page**  **Number/Line**  **Number** | **Reported on**  **Section/Paragraph** |
| --- | --- | --- | --- | --- |
| **Title and abstract** | 1 | (*a*) Indicate the study’s design with a commonly used term in the title or the abstract | Page 1/Line 1 and 1-3,21-49 | Title/Abstract |
|  |  | (*b*) Provide in the abstract an informative and balanced summary of what was done and what was found | Page 1-6/Line 21-49, 91-169,286-295 | Abstract/ Material and methods, Results and Conclusion |
| Introduction | | |  |  |
| Background/rationale | 2 | Explain the scientific background and rationale for the investigation being reported | Page 2/Line 54-90 | Introduction |
| Objectives | 3 | State specific objectives, including any prespecified hypotheses | Page 2/Line 84-90 | Introduction |
| Methods | | |  |  |
| Study design | 4 | Present key elements of study design early in the paper | Page 2,3/Line 91-114 | Material and Methods/ Study area and sampling design |
| Setting | 5 | Describe the setting, locations, and relevant dates, including periods of recruitment, exposure, follow-up, and data collection | Page 3/Line 115-131 | Material and Methods/ Data collection |
| Participants | 6 | (*a*) Give the eligibility criteria, and the sources and methods of selection of participants | Page 3/91-114, | Material and Methods/ sampling design and data collection |
| Variables | 7 | Clearly define all outcomes, exposures, predictors, potential confounders, and effect modifiers. Give diagnostic criteria, if applicable | Page 4-7/Line 131-169, | Material and Methods/ Data collection and statistical analyses  Results/ Laboratory findings |
| Data sources/ measurement | 8* | For each variable of interest, give sources of data and details of methods of assessment (measurement). Describe comparability of assessment methods if there is more than one group | Page 4-7/Line 135-147  Page 7/Line 153-169 | Material and Methods/ statistical analyses Results /Analysis of risk factors |
| Bias | 9 | Describe any efforts to address potential sources of bias | Page4,5/Line 91-114 | Material and Methods/ Study area and sampling design |
| Study size | 10 | Explain how the study size was arrived at | Page 4,5/Line 91-114 | Material and Methods/ Study area and sampling design |
| Quantitative variables | 11 | Explain how quantitative variables were handled in the analyses. If applicable, describe which groupings were chosen and why | Page4-6/Line 109-131 | Material and Methods/ Data collection |
| Statistical methods | 12 | (*a*) Describe all statistical methods, including those used to control for confounding | Page 4-7 / Line 109-170 | Material and Methods/ Analysis of risk factors for agents seropositivity |
|  |  | (*b*) Describe any methods used to examine subgroups and interactions | Page 4,5 / Line 136-147 | Material and Methods/ Analysis of risk factors for agents seropositivity |
|  |  | (*c*) Explain how missing data were addressed | Page 4 / Line 142-145 - there was no missing data | Material and Methods/ Analysis of risk factors for agents seropositivity |
|  |  | (*d*) If applicable, describe analytical methods taking account of sampling strategy | Page 3,4 / Line 135-147 | Material and Methods/ Analysis of risk factors for agents seropositivity |
|  |  | (*e*) Describe any sensitivity analyses | Page 4 / Line 143-147 | Material and Methods/ Analysis of risk factors for agents seropositivity |
| Results | | |  |  |
| Participants | 13* | (a) Report numbers of individuals at each stage of study—eg numbers potentially eligible, examined for eligibility, confirmed eligible, included in the study, completing follow-up, and analysed | Page 7,8 / Line 171-195 | Results |
|  |  | (b) Give reasons for non-participation at each stage | NA | - |
|  |  | (c) Consider use of a flow diagram | - | - |
| Descriptive data | 14* | (a) Give characteristics of study participants (eg demographic, clinical, social) and information on exposures and potential confounders |  | Figures / Fig.1  Tables / Table1-4 |
|  |  | (b) Indicate number of participants with missing data for each variable of interest | NA- there was no missing data |  |
| Outcome data | 15* | Report numbers of outcome events or summary measures | Page 7,8 / Line 171-195 | Results |
| Main results | 16 | (*a*) Give unadjusted estimates and, if applicable, confounder-adjusted estimates and their precision (eg, 95% confidence interval). Make clear which confounders were adjusted for and why they were included |  | Figures / Fig.1  Tables / Table 1-4 |
|  |  | (*b*) Report category boundaries when continuous variables were categorized | - |  |
|  |  | (*c*) If relevant, consider translating estimates of relative risk into absolute risk for a meaningful time period | - |  |
| Other analyses | 17 | Report other analyses done—eg analyses of subgroups and interactions, and sensitivity analyses | Page 7/ Line 152-169 | statistical analyses |
| Discussion | | |  |  |
| Key results | 18 | Summarise key results with reference to study objectives | Page 9-12 / Line 197-284 | Discussion |
| Limitations | 19 | Discuss limitations of the study, taking into account sources of potential bias or imprecision. Discuss both direction and magnitude of any potential bias | Page 11-12/ Line 258-284 | Discussion, Conclusion |
| Interpretation | 20 | Give a cautious overall interpretation of results considering objectives, limitations, multiplicity of analyses, results from similar studies, and other relevant evidence | Page 9-12 / Line 197-284 | Discussion |
| Generalisability | 21 | Discuss the generalisability (external validity) of the study results | Page 9-12 / Line 197-284,286-295 | Discussion, Conclusion |
| Other information | | |  |  |
| Funding | 22 | Give the source of funding and the role of the funders for the present study and, if applicable, for the original study on which the present article is based | Page 18/ Line 435-437 | Funding |

*Give information separately for exposed and unexposed groups.

**Note:** An Explanation and Elaboration article discusses each checklist item and gives methodological background and published examples of transparent reporting. The STROBE checklist is best used in conjunction with this article (freely available on the Web sites of PLoS Medicine at http://www.plosmedicine.org/, Annals of Internal Medicine at http://www.annals.org/, and Epidemiology at http://www.epidem.com/). Information on the STROBE Initiative is available at [www.strobe-statement.org](http://www.strobe-statement.org/).
